# Nursing MSc Theses: A Study of an Iranian College of Nursing and Midwifery in Two Decades (1990-2010)

**DOI:** 10.5539/gjhs.v6n5p118

**Published:** 2014-05-16

**Authors:** Mohadeseh Motamed-Jahromi, Seyedeh Leila Dehghani

**Affiliations:** 1 Department of Medical and Surgical Nursing, School of Nursing, Fasa University of Medical Sciences, Fasa, Iran; 2 Department of Public Health, Behbahan Faculty of Medical Sciences, Behbahan, Iran

**Keywords:** thesis, Iran, nursing, publishing, article

## Abstract

**Aim:**

A thesis is an important part of nursing graduate students’ education, which is also their first systematic and scientific attempt to learn the ABCs of research. Articles derived from theses are important for the dissemination of science and the improvement of nursing as a field. Therefore, it is the goal of the present research is to analyze the different aspects of nursing MSc theses and the number of published articles derived from them.

**Methods:**

This was a descriptive research carried out on 145 nursing MSc theses defended in Razi Faculty of Nursing and Midwifery in Kerman between 1990 and 2010. All of the extracted data were put into an Excel file (2007 version) followed by a data analysis.

**Results:**

The results of this study were then presented via the use of descriptive statistics and figures. The research findings showed that most of the theses used a descriptive or analytical-descriptive method, and 42% of them had patients as their participants. They were usually delivered on the subject of health care, and only 58 articles were extracted from the whole 145 theses.

**Conclusion:**

The process of writing nursing MSc theses and thesis research articles is improving gradually. However, there is a growing need for empirical and semi-empirical research to bridge the gap between theory and practice, which is also a major concern among nurses.

## 1. Introduction

Nowadays, research is a rudimentary factor in the progress of science and the development of different countries. Therefore, more and more attention is addressed to scientific research (Ramos, Padilla, Masia, & Gutierrez, 2008). The scientific production and the amount of research delivered in a country show the degree of scientific development of that country (Borić, 2006; Malekzadeh, Mokri, & Azarmina, 2001).

In the field of medicine, most experiments are carried out in the form of theses. This is because theses have an important role in the formation of scientific and medical knowledge. They are a comprehensive report of the research undergone by students in the final stages of their education. Therefore, the analysis of theses and research projects is of prime importance for universities, by the help of which they can improve the quality of MSc theses (Regojo et al., 2004).

The basic goal of writing an MSc thesis is to strengthen students’ sense of researching and to prepare them to use different research methodologies. In the long term, research can improve their ability to provide solutions for different problems. It can also develop their independence and increase their critical thinking to interpret their findings in more consistently and systematically (Ogunyemi et al., 2005).

The publication of an article in a journal is the best way for the evaluation of its quality. The publication of thesis articles also shows the integration of research and teaching in developed societies (Hren et al., 2004). Publication is a means of the dissemination of knowledge and research findings for scientists all around the world (Dhaliwal & Kumar, 2008), and it serves two major ends, first, to spread new findings and second to evaluate the quality of a research project (Nieminen et al., 2007; Riordan, 2000). Moreover, it proves the professional and scientific authority of a researcher and the organization in which he/she works (Nieminen et al., 2007; Salmi, Gana, & Mouillet, 2001; Younes, Deheinzelin, & Birolini, 2005).

An MSc thesis is no place for mere creativity (Younes, Deheinzelin, & Birolini, 2005), and nor is it only for the fulfillment of Master degree. A thesis should be delivered on a publishable subject (Hyderi, 2006). It is necessary for researchers to publish articles to have an academic occupation; therefore they should choose only those titles and themes that are more likely to be published (Hyderi, 2006; Nieminen et al., 2007). As a matter of fact, the publication of thesis articles is significantly influenced by the advocating role of research supervisors (Cooper & Turpin, 2007; Polasek, Kolcic, Buneta, Cikes, & Pecina, 2006). They are responsible for encouraging and guiding graduate students to publish their articles, which consequently leads to their professional and academic development (Singhi et al., 2007).

Nursing is an important field of medical science and health systems. It plays a key role in improving the life quality and health of individuals. Just like all other fields of science, nursing can be developed through research, thus nursing theses are considered as an important source of knowledge production.

Each year, a great number of graduate students and professors participate in writing dissertations. It goes without saying that enough care should be taken in writing a thesis and increasing its quality. This affects the process of knowledge production and the condition of nursing in a society. As a result of research and knowledge accumulation, medical services are improved exponentially, and health system will become under constant progression. The high quality of research delivered in the field of medicine could guarantee the advancement of these services. Prior studies have shown the fact that the quality of theses is on the rise. For example, Davami et al. demonstrated that the quality of thesis research projects has increased in comparison with former theses (Davami, Moiini, & Rafiei, 2001).

A quick literature review signifies that researchers in other fields of science often focus on theses carried out in fulfilling different degrees (e.g. PhD, MSc or BSc), but in medicine, general practitioners’ dissertations have been under the spotlight. For instance, Eftekhari, Rezaeian, Zare Bidaki and Arabshahi (2013) in Iran analyzed the dissertations of Rafsanjan^Note 1^ Medicine College in 2012. They indicated that the number of articles extracted from the dissertations is not that bad, but not exactly close to the ideal (Eftekhari et al., 2013). A research done by Nieminen et al. in Finland in 2007 reveals that the number of published articles derived from the dissertations is less than the whole dissertations (Nieminen et al., 2007). Singhi et al. stated that dissertation is a good means for publishing research articles by students and professors. They also reported that the omission of dissertation as a course in India resulted in a considerable drop in the number of their published articles (Singhi et al., 2007).

Presently, there are 29 colleges of Nursing and Midwifery in Iran admitting MSc students of nursing. One of the oldest colleges is Razi Nursing and Midwifery College, which is a branch of Kerman University of Medical Sciences and is located in the southeast of Iran. It was established in 1964, and the admission MSc and PhD students respectively began in 1990 and 2006, and it is still in progress. Due to the extremely vital role of nursing students’ theses in the dissemination of knowledge and the development of the field, we decided to analyze the theses of Razi College since from the beginning of its MSc programs. We will attempt to scrutinize the number of published articles derived from these theses as well as the factors contributing to the publication of these articles. It should be noted that very few studies have formerly addressed this issue in Iran.

## 2. Methodology

This is an analytical-descriptive research using census method to extract data from 145 nursing MSc theses of Razi Nursing and Midwifery College. The theses were done between 1990 and 2010. The original texts of the theses were also analyzed to gather the data. Other relevant information such as students’ gender and age, the year, location, advisor, supervisor and colleagues was provided. We also took heed of the thesis subject, methodology (descriptive, analytical, empirical, etc.), participants as well as the number of published articles derived from each thesis and the name of the journals in which the articles were published. To determine the number of published articles, we referred to three Iranian data bases including SID, IranMedex and Magiran and three international databases which were Google Scholar, PubMed and ISI. The data were put into an excel file (2007 version) with the purpose of analysis. The results were then presented via the use of descriptive statistics and relevant figures.

This research obtained the official consent of the head of Razi Nursing and Midwifery College and the ethics committee. The results are presented without stating the names of the students and professors.

## 3. Results

**Figure 1:**
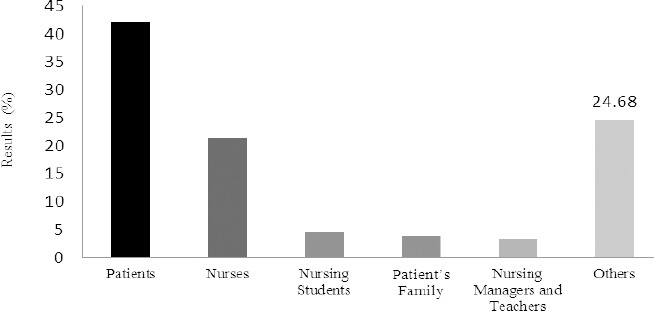
Target population

From the 145 MSc theses defended between 1990 and 2010, 104 theses (71%) were done by female students and 41 theses (28.2%) were done by male students. 128 theses (88%) were done under the aegis of only one supervisor. According to Figure 1, 65 theses (42%) focused on patients as their target. The target population of the theses was analyzed between 1990 and 2010. Nursing instructors and managers received the least attention from the researches; they comprised only 3.25% of the whole 145 theses.

**Figure 2:**
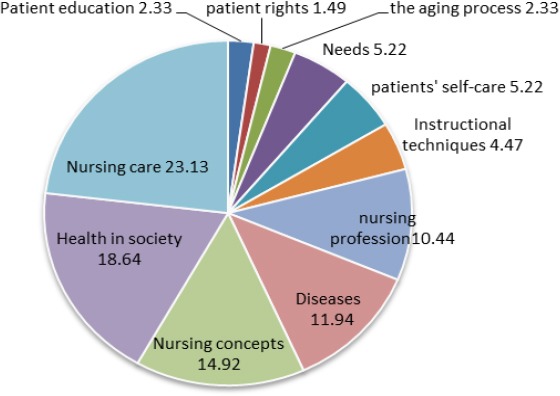
Subject of theses (%)

Most of the theses (23.13%) were delivered on the subject of nursing care (e.g. the quality of care, the use of care methods, the satisfaction of patients, indoor health care). According to Figure 2, the next most frequent subjects were respectively health in society (e.g. disease prevention, personal hygiene, family health, work health), nursing concepts (e.g. pain, stress, mental images, sleep, health), diseases, nursing profession, patients’ self-care etc. The least frequent subjects were about patient rights (e.g. consent, nurses and nursing managers’ understanding of patient rights).

**Figure 3:**
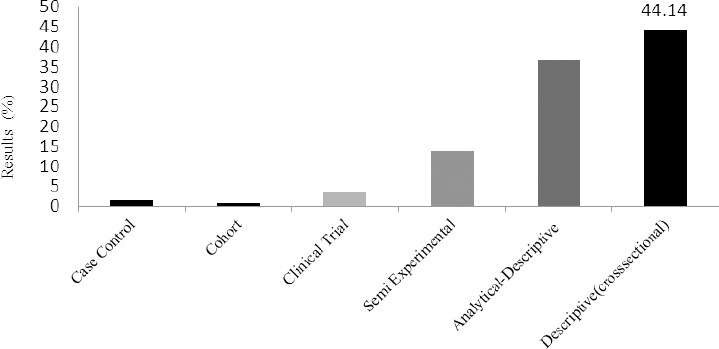
Type of studies

According to Figure 3, 44.14% of the theses during the two decades were of descriptive type (cross-sectional). The rest of the research types respectively included analytical-descriptive, semi-empirical, clinical, cohort and case control studies.

**Figure 4:**
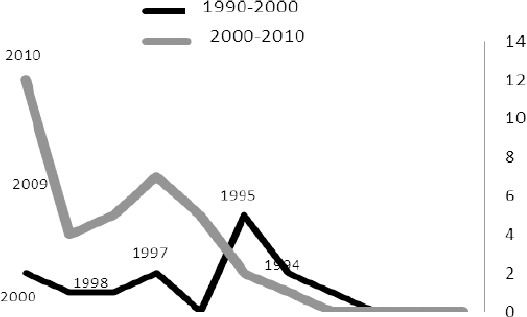
Number of articles

The findings of the study also showed that 58 articles derived from the theses were published in Iranian and international journals between 1990 and 2010, and year 2010 had the highest number of published articles (Figure 4).

We found that the average distance between the defense and the article publication was 3.01 years. This time slot ranged from 1 to 15 years. Some of the articles were also published in the same year in which they were defended. Most of the articles (98%) were written in Persian and were published in Scientific-Research^Note 2^ journals. The number of published articles extracted from one thesis was at least 1 and at most 3. The average number of authors for a single article was 3, and the name of student, supervisor and advisor were cited in all of the articles. The student was the main author or the responsible author on most of the papers. From the 58 published articles, 4 articles (0.04%) had ISI index.

## 4. Discussion

The MSc students’ first step toward research and scientific development is their attempt to write their thesis and publish its findings. The analysis of research activities, thesis writing and scientific publication of a university is a valuable means of recognizing the scientific status of the students. In other words, theses are representative of the scientific condition of researchers. Theses cannot be modified or substituted after their publication. Hence, the correct and regular presentation of theses as a scientific report of a research project is of prime importance.

The findings of the present research proved that 80% of the theses analyzed in this study used a descriptive or analytical-descriptive method, and only 19% exploited an empirical, semi-empirical, cohort or case control method. The merit of using a descriptive method is due to its practicality and ease of implementation. Using this method, a researcher is better able to deal with the actual problems he/she faces. A research carried out in the US in 1991 reported that more than half of the research projects were descriptive and only 17% of them were clinical trials, which is in line with the findings of our study (Fowkes, Garraway, & Sheehy, 1991).

Based on the results, the recurrent theme of the theses revolved around the issue of nursing care, health in society and nursing concepts. The chosen subjects could also indicate why descriptive method has been so popular among the researchers. Considering the fact that in any university the thesis themes can potentially show the research trajectory of that university, it can be predicted that in Razi College of Nursing and Midwifery, the most dominating themes are health care and concepts, and disease prevention.

The number of published articles (58 articles) were less than the overall number of defended theses (145 theses), which is indicative of the students and professors’ lack motivation in publishing the results of their projects. Other studies also approve the fact that although publishing articles are fruitful, forcing students to publish their research findings is a universal problem (Nieminen et al., 2007; Salmi et al., 2001; Younes, Deheinzelin & Birolini, 2005). However, the lack of publication could be also ascribed to the fact that some subjects are not welcomed by scientific journals. Another problem is that if the findings of a thesis are not published on time, after some time they might be considered stale and outdated since other studies might supersede them. Some studies have argued that the lack of publication of articles in comparison with the overall number of theses is because of the researchers’ high work load (i.e. their education and routine profession), financial limitations and the lack of necessary instruments (Nieminen et al., 2007; Polasek, Kolcic, Buneta, Cikes, & Pecina, 2006).

The number of published articles in the first decade (i.e. 1990-2000) was relatively less than that of the second decade (i.e. 2000-2010). From the author’s perspective, this can be due to the following reasons: the lack of students’ knowledge with respect to writing research articles, the less importance of knowledge dissemination, the existence of fewer relevant scientific journals, stricter review policies, manual submission of articles and the lack of access to internet.

More articles were published in the 2000s especially in the year 2010. Many factors account for this rise in the number of published articles. During this decade more workshops and courses were hold on writing and publishing research papers, which as a result led to the increase of students’ understanding and appreciation of valid scientific journals. Students and professors also became aware that the publication of research articles lays a foundation for their future profession and academic status. More journals came into being (especially nursing specialized journals), and the growth of internet came in handy with respect to the easy submission of articles. The competition between students and professors to publish their findings grew even fiercer, and consequently, a considerable surge in the number of published articles emerged. It is also interesting to mention that recently in Kerman University of Medical sciences, theses are considered as research projects, in which supervisors play an executive role, and advisors and students are colleagues. Each project needs to be approved by the faculty in a process which includes arbitration, contracting, development report and final report. Publishing research articles yields positive grades, which is also a very important factor in the increase of the published articles during this decade.

The results also showed that the average distance between thesis defense and the article publication is 3.01 years which is almost approved by Eftekhari’s et al. study in 2013. They carried out a research focusing on the theses of Rafsanjan Medicine College. They found that the average distance between the defense and the article publication is 2.9 years (Eftekhari, Rezaeian, Zare Bidaki, & Arabshahi, 2013). A French research reports that 27% of theses were published in the first year and almost 50% of them were published after the second year (Salmi et al., 2001). It is notable that this distance has relatively decreased during the recent years, which can be simply ascribed to the rule^Note 3^ that publishing thesis articles comprise a part of the total thesis grade.

## 5. Conclusion

After analyzing the nursing MSc theses of Kerman Nursing College during a 20-year period, it can be concluded that the nursing theses and the articles derived from them are gradually developing in terms of quality and quantity. It is necessary to note that planning and investment are two essential elements when it comes to carrying out empirical and semi-empirical studies. Such studies could bridge the gap between theory and practice which is also a critical issue among nurses. The realization of this goal demands holding short courses on empirical research, creating the corresponding background and providing necessary instruments.

## Notes

**Notes**

Note 1. Rafsanjan is a town located in the province of Kerman in Iran.

Note 2. The Iran Scientific-Research journals are ranked as the most valid and reliable journals.

Note 3. In Kerman University of Medical Sciences, to get a complete grade, it is mandatory to publish a paper extracted from the thesis.

## References

[ref1] Borić V. (2006). Bibliometric Analysis of the Articles from the School of Dental Medicine, University of Zagreb, Indexed in Web of Science Database. Acta Stomatol Croat (Online).

[ref2] Cooper M., Turpin G. (2007). Clinical psychology trainees’ research productivity and publications: An initial survey and contributing factors. Clinical Psychology & Psychotherapy.

[ref3] Davami M. H., Moiini L., Rafiei M. (2001). Evaluation of principles consideration in the dissertations of medical students submitted at Arak university of medical sciences from 1373 (1994) to 1379 (2000). Arak Medical University Journal (AMUJ).

[ref4] Dhaliwal U., Kumar R. (2008). An observational study of the proceedings of the All India Ophthalmological Conference, 2000 and subsequent publication in indexed journals. Indian journal of ophthalmology.

[ref5] Eftekhari Y., Rezaeian M., Bidaki Z., Arabshahi M. (2013). A Survey on the Status of Article Publication from Defended Medical Theses in Rafsanjan University of Medical Sciences, School of Medicine during 1993-2007. Journal of Rafsanjan University of Medical Sciences.

[ref6] Fowkes F. G., Garraway W. M., Sheehy C. K. (1991). The quality of health services research in medical practice in the United Kingdom. Journal of Epidemiology and Community Health.

[ref7] Hren D., Lukic I. K., Marusic A., Vodopivec I., Vujaklija A., Hrabak M., Marusic M. (2004). Teaching research methodology in medical schools: Students’ attitudes towards and knowledge about science. Medical Education.

[ref8] Hyderi A. (2006). PG thesis: Idealistic vs realistic. Indian Journal of Pediatrics.

[ref9] Malekzadeh R., Mokri A., Azarmina P. (2001). Medical science and research in Iran. Arch Iran Med.

[ref10] Nieminen P., Sipila K., Takkinen H.-M., Renko M., Risteli L. (2007). Medical theses as part of the scientific training in basic medical and dental education: Experiences from Finland. BMC Medical Education.

[ref11] Ogunyemi D., Bazargan M., Norris K., Jones-Quaidoo S., Wolf K., Edelstein R., Calmes D. (2005). The development of a mandatory medical thesis in an urban medical school. Teaching and Learning in Medicine.

[ref12] Polasek O., Kolcic I., Buneta Z., Cikes N., Pecina M. (2006). Scientific production of research fellows at the Zagreb University School of Medicine, Croatia. Croatian Medical Journal.

[ref13] Ramos J. M., Padilla S., Masia M., Gutierrez F. (2008). A bibliometric analysis of tuberculosis research indexed in PubMed, 19972006. The International Journal of Tuberculosis and Lung Disease.

[ref14] Regojo Z. O., Lamata H. F., Sánchez Z. J. M., Elizadet B. A., Navarro G. J., Valdivia U. J. G. (2004). Quality analysis of the statistical used resources (material and methods section) in thesis projects of a university department. Actas urologicas espaolas.

[ref15] Riordan F. A. I. (2000). Do presenters to paediatric meetings get their work published?. Archives of Disease in Childhood.

[ref16] Salmi L. R., Gana S., Mouillet E. (2001). Publication pattern of medical theses, France 1993-98. Medical Education.

[ref17] Singhi S., Rajagopal R., Mehta Ludhiana R., Nirmalan P., Hyderi A., Ravikiran S. (2007). PG Thesis: Idealistic vs realistic. Indian Journal of Pediatrics.

[ref18] Younes R. N., Deheinzelin D., Birolini D. (2005). Graduate education at the faculty of medicine of the University of Sao Paulo: quo vadis?. Clinics.

